# Establishment of an *Ex Vivo* Inflammatory Osteoarthritis Model With Human Osteochondral Explants

**DOI:** 10.3389/fbioe.2021.787020

**Published:** 2021-12-21

**Authors:** Kaihu Li, Penghui Zhang, Yong Zhu, Mauro Alini, Sibylle Grad, Zhen Li

**Affiliations:** ^1^ Department of Orthopaedics, Xiangya Hospital of Central South University, Changsha, China; ^2^ AO Research Institute Davos, Davos, Switzerland; ^3^ Department of Orthopaedic Surgery, The Seventh Affiliated Hospital, Sun Yat-sen University, Shenzhen, China

**Keywords:** osteoarthritis, *ex vivo*, model, osteochondral explant, inflammation, human

## Abstract

Osteoarthritis (OA) is the most common degenerative joint disease without clear pathophysiological mechanism and effective drugs for treatment. Although various animal models exist, the translation of the outcome into clinics remains difficult due to species differences. In this study, an *ex vivo* inflammatory OA model was induced using different concentrations of interleukin one beta (IL-1*β*) and tumor necrosis factor *α* (TNF-α) on explants from the human femoral head. In the inflammatory OA groups, the gene expression levels of cartilage catabolism (matrix metalloproteinase 1 (MMP1), matrix metalloproteinase 3 (MMP3)), and inflammation (interleukin 6 (IL-6), interleukin 8 (IL-8)) markers were significantly upregulated, while the anabolic genes (collagen 2 (COL2), aggrecan (ACAN), and proteoglycan 4 (PRG4)) were downregulated compared to the control group. The release of cytokines (IL-6, IL-8) and nitric oxide (NO) in the conditioned medium was also upregulated in inflammatory OA groups. The Safranin O/Fast Green staining showed loss of proteoglycan in the superficial zone cartilage after cytokine treatment. The results indicated that an *ex vivo* inflammation and degeneration model was successfully established using osteochondral explants from the human femoral head. This model can be used to elucidate the in-depth mechanism of inflammatory OA and to screen new drugs for OA treatment.

## Introduction

Osteoarthritis (OA) is the most common degenerative joint disease, mainly invading knee and hip joints (Martel-Pelletier et al., 2016). Its prevalence and incidence keep rising year by year, leading to increasing burden in most countries (Safiri et al., 2020; Peat and Thomas, 2021). OA mainly manifests progressive and recurrent joint pain, swelling, and restricted activity causing many years lived with disability (YLDs) (Safiri et al., 2020). The underlying pathophysiologic mechanism has not been fully elucidated due to the heterogeneity of the disease and multiple risk factors like trauma, genetics, gender, and aging (Loeser et al., 2016; He et al., 2020; Coryell et al., 2021). Furthermore, there are no effective disease-modifying OA drugs (DMOADs) approved yet, other than medicines for relieving symptoms (Cai et al., 2021; Oo et al., 2021).

To elucidate the pathological mechanism and screen the effectiveness of new drugs, numerous *in vitro* and *in vivo* OA models have been developed (Teeple et al., 2013; McCoy, 2015; Johnson et al., 2016). *In vitro* cell culture models and cytokines, such as interleukin one beta (IL-1*β*) and tumor necrosis factor *α* (TNF-α), have widely been used to establish OA-like conditions, since low-grade inflammatory circumstances are known to play a major regulatory role in the disease progression (Robinson et al., 2016). However, isolated *in vitro* cultured cells lack the environment of cells embedded in their native extracellular matrix. For *in vivo* studies, various animals and surgical methods have been used to establish a destabilized OA model, simulating posttraumatic OA (Teeple et al., 2013; McCoy, 2015). Nonetheless, the translation of animal data to clinical practice is still challenging due to significant variations between animals and humans.

To overcome these limitations and better reflect real circumstances of human OA progression, *ex vivo* explant models have great potential, as they are also in accordance with the 3Rs principle of animal testing: replace, reduce, refine (Schwab et al., 2017; Cope et al., 2019; Humpenoder et al., 2021). Geurts et al. (Geurts et al., 2018) have established an inflammatory OA model induced by lipopolysaccharide with osteochondral plugs from human OA knee and spine joints to assess the effects of potential drugs. The tissue viability of explants from the human OA tibia plateau has been proven stable within a period of at least 2 or 4 weeks (Kleuskens et al., 2021). In addition, different OA-like phenotypes have been established with human osteochondral explants, such as loading-induced models, inflammatory models, and hypertrophy subtypes (Houtman et al., 2021). To date, no study has been reported that comprehensively characterized the *ex vivo* inflammatory OA model with human samples induced with different concentrations of IL-1*β* and TNF-α in combination.

In this study, we aimed to establish and characterize an *ex vivo* inflammatory OA model using osteochondral explants isolated from human femoral heads. The effects of IL-1β and TNF-α at different doses were investigated. The expression of genes related to cartilage anabolism, catabolism, and inflammation was evaluated by quantitative real-time polymerase chain reaction (qRT-PCR). The glycosaminoglycan (GAG), nitric oxide (NO), and inflammatory cytokine releases into the conditioned medium were quantified. In addition, the cell viability and tissue morphology of explants were assessed by live cell staining and histological methods.

## Materials and Methods

### Osteochondral Explant Isolation

**FIGURE 1:**
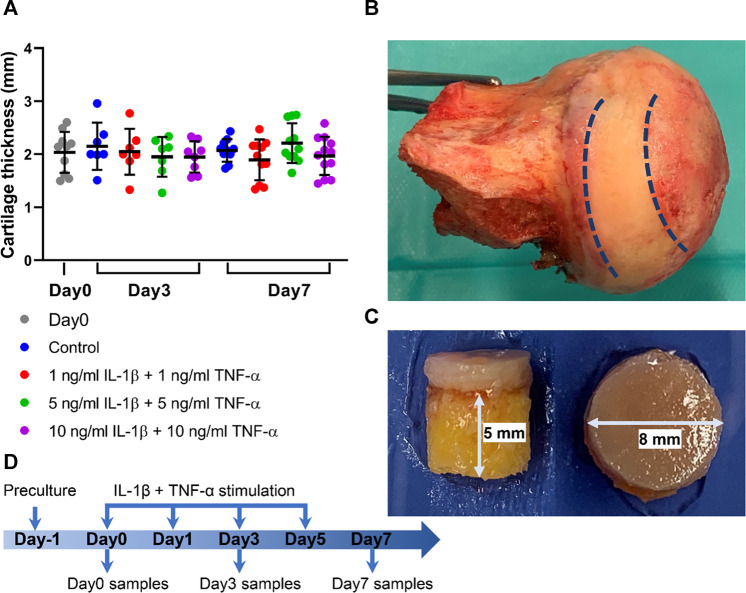
Osteochondral explants from human femoral heads for establishment of the *ex vivo* inflammatory OA model. **(A)** Cartilage thickness of explants from the Day0 group, control group, and groups stimulated by different concentrations of IL-1β and TNF-α for 3 and 7 days (*n* = 7–12). **(B)** Photograph of a human femoral head. **(C)** The height of subchondral bone and diameter of explant harvested from the areas between the dotted line in (B). (D) Experimental design of the inflammatory OA *ex vivo* model. Statistical analysis was performed by one-way analysis of variance (ANOVA). Data presented as means ± SD.

Osteochondral explants were harvested from human femoral heads of patients who underwent hip replacement operation with their informed consent and approval by the cantonal ethical commission (KEK-ZH-NR: 2010-0444/0). The clinical characteristics of donors and the Kellgren–Lawrence classification (Kellgren and Lawrence, 1957) of hip joints used in this study are summarized in Table 1. The average age of included patients was 75.75 ± 11.10 years (range from 59 to 89 years). Osteochondral explants (Figure 1B) were isolated from the non-tissue wear regions of the femoral heads using a trephine with an inner diameter of 8 mm. The subchondral bone was cut with a circular saw to reach a height of 5 mm (Figure 1C). After isolation, the explants were rinsed in phosphate-buffered saline (PBS) three times before culture.

**TABLE 1:** Demographic characteristics of patients included.

No	Gender	Age (years)	Kellgren–Lawrence grade	No. of explants isolated
1	Male	86	2	13
2	Female	81	4	9
3	Male	89	3	11
4	Female	70	3	9
5	Female	76	2	14
6	Female	83	3	10
7	Female	59	3	12
8	Female	62	4	8

### Experimental Design

All explants were first cultured for 1 day at 37°C, 5% CO_2_, and 80% humidity in 12-well nontreated plates with 2 ml explant medium per well, which consisted of high-glucose DMEM medium (Gibco, Grand Island, NY, USA), supplemented with 100 μg/ml sodium pyruvate (Sigma-Aldrich, St. Louis, MO, USA), 2.5% HEPES (Gibco), 1% nonessential amino acids (Gibco), 50 μg/ml ascorbic acid (Sigma-Aldrich), 1% ITS + Premix (Corning, Tewksbury, MA, USA), 100 U/ml penicillin, and 100 μg/ml streptomycin. After 1-day preculture, one explant from each donor was processed for further analysis as the normalization baseline. Other explants were randomly divided into four groups and respectively cultured in 1) explant medium (Control), 2) explant medium plus 1 ng/ml IL-1β (PeproTech) + 1 ng/ml TNF-α (R&D Systems, Minneapolis, MN, USA), 3) explant medium plus 5 ng/ml IL-1β + 5 ng/ml TNF-α, or 4) explant medium plus 10 ng/ml IL-1β + 10 ng/ml TNF-α; explants were harvested on day 3 and day 7. The conditioned medium was collected on day 0, day 1, day 5, and day 7 for further analysis (Figure 1D).

### GAG Measurement

The release of sulfated GAG into the conditioned medium was measured with 1,9-dimethyl-methylene blue (DMMB, Sigma-Aldrich, St. Louis, MO, USA) color reagent. Chondroitin sulfate was used to generate the standard curve (concentration range 0–2.5 μg/50 μl). Briefly, 50-μl standards or samples were added into each well followed by 200 μl DMMB reagent. Absorbance at 535 nm was immediately read by a Tecan microplate reader.

### NO Measurement

NO in the conditioned medium was measured by Griess Reagent System (Promega Corporation, Madison, WI, USA). Nitrite was used to generate the standard curve with the maximum concentration of 100 μM. Firstly, 50-μl standards or samples were pipetted into the plate. Secondly, 50 μl Sulfanilamide Solution and NED Solution was successively added into each well with each incubated for 10 min at room temperature and protected from light. The absorbance at 535 nm was read by a microplate reader.

### Enzyme-Linked Immunosorbent Assay

The protein concentrations of human IL-6 and IL-8 in the conditioned media were assessed by ELISA (R&D Systems) according to the manufacturer’s instructions.

### Gene Expression Analysis

The full-thickness cartilage tissue was removed from subchondral bone and then minced into small pieces and digested with 2 mg/ml pronase (Roche, Basel, Switzerland) for 2 h at 37°C as described in a previous study (Caprez et al., 2018). Then, cartilage fragments, precooled with liquid nitrogen, were pulverized before transferring into tubes with TRI Reagent (Molecular Research Center, Cincinnati, OH, USA). The pulverized cartilage tissue within TRI was then homogenized with a tissue lyser (Retsch). After centrifugation, the supernatant was transferred into fresh tubes. 1-Bromo-3-chloropropane (BCP, Sigma-Aldrich) was then added into each tube. After 15 min of shaking and 15 min of centrifugation, the aqueous phase containing RNA was transferred into fresh tubes. RNA was then purified with RNeasy Mini Kit (Qiagen, Hilden, Germany). The RNA concentration was measured by a NanoDrop spectrophotometer (Thermo Fisher Scientific Inc., Waltham, MA, USA). Reverse transcription was conducted with SuperScript Vilo RT Kit (Thermo Fisher Scientific). PCR to assess gene expression was performed with QuantStudio Flex 7.0 instrument and TaqMan Gene Expression Master Mix (Applied Biosystems, Foster City, CA, USA). The primer and probe sequences of tested genes are listed in Table 2. RPLP0 ribosomal RNA was used as endogenous control. Data were analyzed using the 2^−ΔΔCt^ method.

**TABLE 2:** Oligonucleotide primers and probes used for qPCR.

Gene	Primer and probe	Sequence/catalog number
RPLP0	Forward primer (5′→3′)	TGG GCA AGA ACA CCA TGA TG
	Reverse primer (5′→3′)	CGG ATA TGA GGC AGC AGT TTC
	Probe (5′FAM/3′TAMRA)	AGG GCA CCT GGA AAA CAA CCC AGC
COL2	Forward primer (5′→3′)	GGC AAT AGC AGG TTC ACG TAC A
	Reverse primer (5′→3′)	GAT AAC AGT CTT GCC CCA CTT ACC
	Probe (5′FAM/3′TAMRA)	CCT GAA GGA TGG CTG CAC GAA ACA TAC
COL10	Forward primer (5′→3′)	ACG CTG AAC GAT ACC AAA TG
	Reverse primer (5′→3′)	TGC TAT ACC TTT ACT CTT TAT GGT GTA
	Probe (5′FAM/3′TAMRA)	ACT ACC CAA CAC CAA GAC ACA GTT CTT CAT TCC
ACAN	Forward primer (5′→3′)	AGT CCT CAA GCC TCC TGT ACT CA
	Reverse primer (5′→3′)	CGG GAA GTG GCG GTA ACA
	Probe (5′FAM/3′TAMRA)	CCG GAA TGG AAA CGT GAA TCA GAA TCA ACT
PRG4		Hs00981633_m1
MMP1		Hs00899658_m1
MMP3		Hs00968305_m1
IL-6		Hs00174131_m1
IL-8		Hs00174103_m1

Primers and probes presented with sequences were custom designed; primers and probes presented with catalogue numbers were from Applied Biosystems.

### Lactate Dehydrogenase/Ethidium Homodimer Staining

Cartilage of explants was removed from subchondral bone with a scalpel, snap frozen in liquid nitrogen, and stored at -20 °C. Sections with thickness of 10 μm were made by cryosection microtome (Leica, Wetzlar, Germany). The lactate dehydrogenase (LDH) solution (10 ml) was prepared as follows (Jahn and Stoddart, 2011): 54 mg lactic acid (Sigma) and 17.5 mg nicotinamide adenine dinucleotide (NAD, Sigma) were added into a beaker and were mixed with 8 ml 40% Polypep solution (Sigma) which was preheated to 37 °C. Then, the pH was adjusted to 8.0 with 10% NaOH and 40% Polypep solution was added to achieve a final volume of 10 ml. Finally, 30 mg nitro blue tetrazolium (NBT, Sigma) was added to the solution before use. After rinsing in deionized water, the sections were incubated in ethidium homodimer (Sigma) for 45 min at room temperature and then incubated in LDH solution at 37°C for 3 h. The sections were then fixed with 4% formalin for 15 min and mounted with an aqueous mounting reagent (Dako, Glostrup, Denmark). In each group, six full-thickness histological slides from three explants were counted, with two inconsecutive sections from each explant. Cells with blue color or combined color (blue and red) were considered as alive, while only reddish-stained cells were considered as dead. The numbers of alive and dead cells were counted with ImageJ software (ImageJ, National Institutes of Health, Bethesda, MD, USA).

### Safranin O/Fast Green Staining

Osteochondral explants were fixed in 4% formalin for 48 h and then decalcified with 12.5% EDTA containing 1.25% NaOH for about 2 weeks. After decalcification was accomplished, explants were dehydrated with ascending series of ethanol in an automatic spin tissue processor (Microm, STP 120-2) and embedded manually with the tissue embedding center (Microm, EC 350-1). Sections with thickness of 5 μm were made with paraffin microtome (Microm, HM 355S). For staining, sections were first deparaffinized with xylene and then hydrated with gradient ethanol and deionized water. Sections were stained in Weigert’s Hematoxylin (Sigma) for 10 min followed by rinsing in tap water for 10 min. The slides were then stained with 0.02% Fast Green (Sigma) for 6 min followed by differentiation with 1% acetic acid (Fluka) for 30 s. Following that, slides were stained with 0.1% Safranin O (Sigma) for 12 min and then rinsed in deionized water. Sections were then differentiated with 70% ethanol for 10 s and mounted with Eukitt (Sigma) after dehydration. In each group, three staining images (n = 3) of cartilage were quantified with one image per donor using ImageJ software. The cartilage on the whole image (Figure 3A) was defined as the total cartilage area. The area on the image (Figure 3A) without red staining was defined as the Safranin O-negatively stained area. The percentage of Safranin O-negatively stained area in each image was calculated (Shi et al., 2019) and then normalized to its individual control sample from the corresponding donor.

### Statistical Analysis

All the statistical analyses were performed with GraphPad Prism 8.4.0 (GraphPad Software, Inc., La Jolla, CA, USA). The Shapiro–Wilk normality test was used to define whether data were normally distributed. One-way analysis of variance (ANOVA) was conducted to determine differences between three or more groups for continuous variables with normal distribution. The Kruskal–Wallis test was conducted to evaluate the differences for non-normally distributed data. *p* < 0.05 was considered as statistical significance.

## Results

### Explant Size

Osteochondral explants with 8-mm diameter were obtained using a trephine device. The height of subchondral bone was cut to reach 5 mm with the circular saw (Figure 1B). After explants were harvested, cartilage thickness was measured with a caliper. The average cartilage thickness in each group was assessed; the thickness reached 2.03 ± 0.36 mm and was comparable among all the groups (Figure 1A).

### Tissue Viability

**FIGURE 2:**
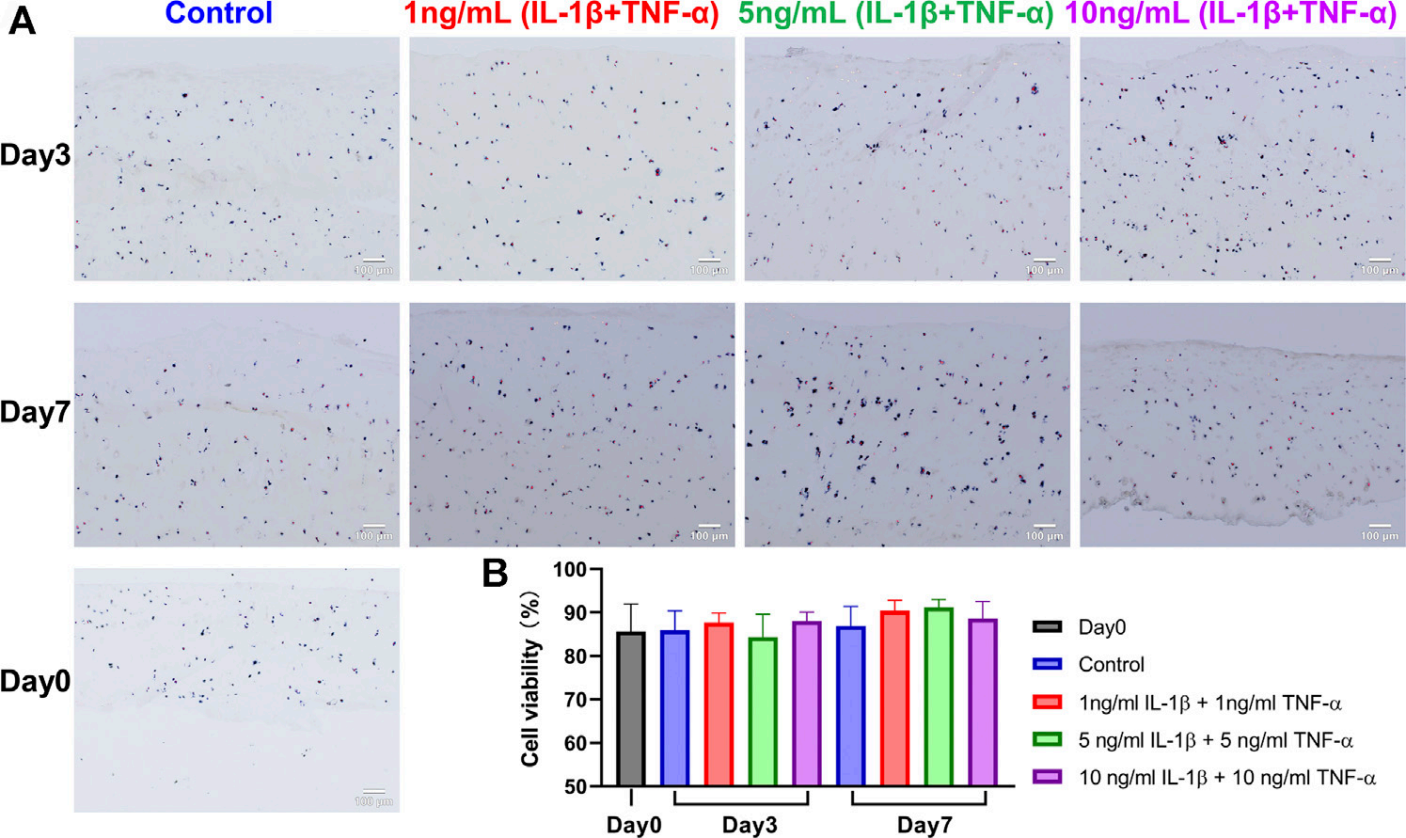
LDH/ethidium homodimer staining on snap-frozen sections of unstimulated and IL-1β- and TNF-α-stimulated osteochondral explants from human femoral heads. **(A)** Representative staining images of cartilage in explants from the Day 0 group, control group, and groups stimulated by different concentrations of IL-1β and TNF-α for 3 and 7 days. Blue and blue/red-stained cells were alive, while red-only-stained cells were dead. Scale bars, 100 μm. **(B)** Quantification analysis of tissue viability among above groups (*n* = 6). Statistical analysis was performed by one-way analysis of variance (ANOVA). Data presented as means +SD.

Cell viability in cartilage tissue of explants was evaluated on cryosections stained with LDH/ethidium homodimer (Figure 2A). Cell viability of the day 0 group was 85.6 ± 6.3%. Data showed that the cell viability was stable during culture and was also unchanged when explants were treated with combined cytokines at different concentrations (Figure 2B).

### Safranin O/Fast Green Staining

**FIGURE 3:**
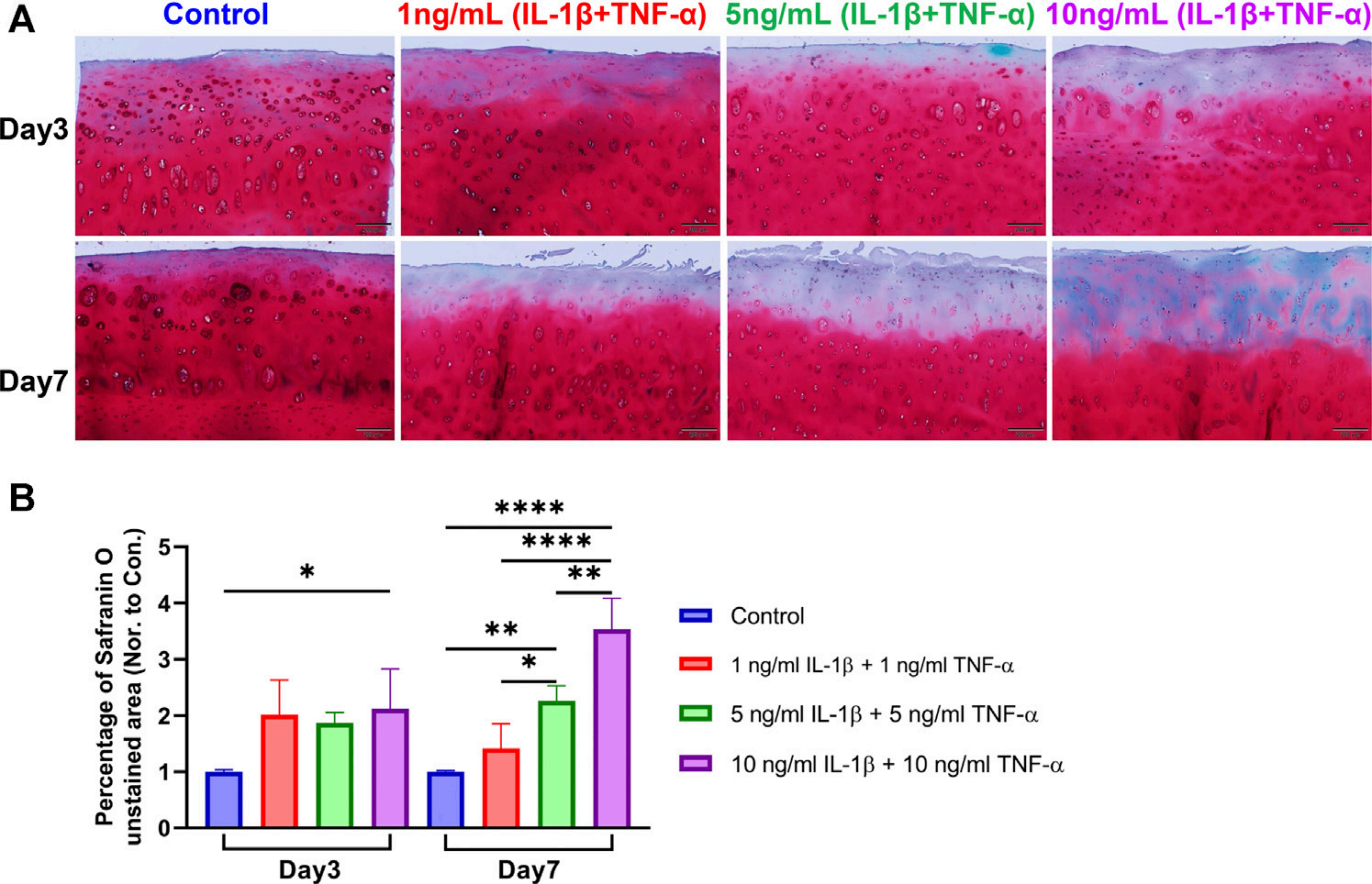
Safranin O/Fast Green staining on sections of unstimulated and IL-1β- and TNF-α-stimulated osteochondral explants from human femoral heads. **(A)** Representative staining images of explants from the control group and groups stimulated by different concentrations of IL-1β and TNF-α for 3 and 7 days. Scale bars, 200 μm. **(B)** Safranin O-unstained area among the above groups was quantified and normalized to the control group of each donor as 1 (*n* = 3). Statistical analysis was performed by one-way analysis of variance (ANOVA). Data presented as means +SD. ns p > 0.05, *p < 0.05, **p < 0.01, ****p < 0.0001.

Proteoglycan distribution in the explants was assessed with Safranin O/Fast Green staining using paraffin sections. In the control group, there was no or little proteoglycan loss, while cytokine-stimulated groups presented mild to severe proteoglycan loss in the cartilage (Figure 3A). Significant differences were observed in the percentage of the Safranin O-negatively stained area between the control group and inflammatory groups on both day 3 and day 7 (Figure 3B). Furthermore, the Safranin O-negatively stained area showed significant differences among different treatment groups on day 7. The histological results indicated that the cytokine stimulation enhanced the loss of proteoglycan in the extracellular matrix of explant cartilage tissue, which is a typical histopathological change in OA cartilage.

### Gene Expressions

**FIGURE 4:**
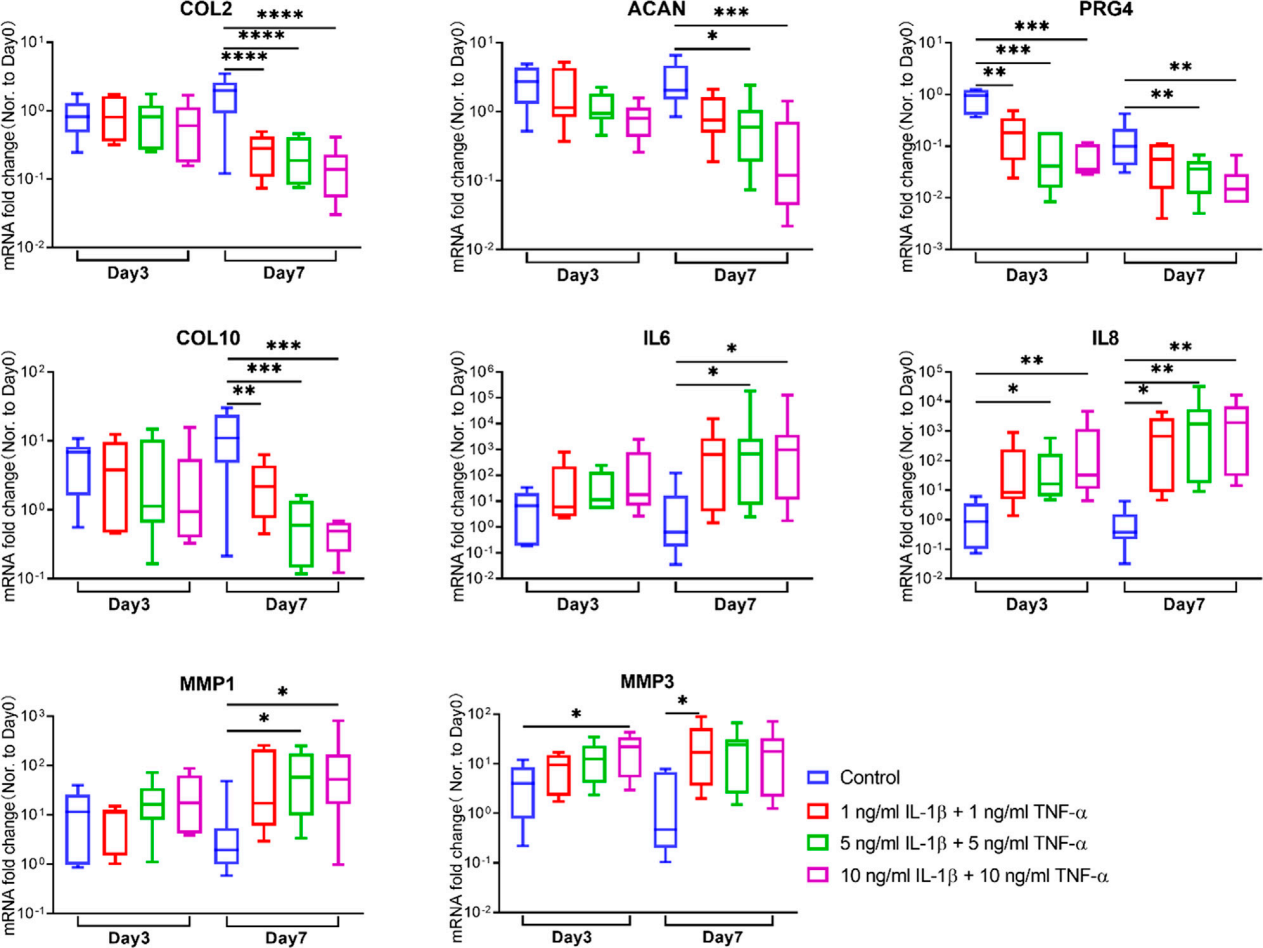
Gene expression changes of chondrocytes in osteochondral explants from human femoral heads stimulated with different concentrations of IL-1β and TNF-α for 3 and 7 days. The relative gene expression levels of COL2, ACAN, PRG4, COL10, IL6, IL8, MMP1, and MMP3 were measured by qRT-PCR (*n* = 6–8). Statistical analysis was performed by one-way analysis of variance (ANOVA) or Kruskal–Wallis test. Data presented as box and whisker plots with boxes indicating the 25th to 75th percentiles, whiskers indicating the maximum and minimum values, and horizontal lines in the boxes indicating medians. *p < 0.05, **p < 0.01, ***p < 0.001, ****p < 0.0001.

The gene expression levels of catabolism, anabolism, and inflammation markers in the cartilage tissue were assessed by qRT-PCR (Figure 4). For catabolic genes, compared with the respective control group at the same time point, MMP1 was markedly elevated in the 5- and 10-ng/ml cytokine-induced groups on day 7, while MMP3 showed remarkable upregulation in the 10-ng/ml inflammatory group on day 3 and the 1-ng/ml group on day 7. As for anabolism genes, COL2 was significantly decreased in all inflammatory groups on day 7, while ACAN was significantly downregulated in the 5- and 10-ng/ml inflammatory groups on day 7. PRG4 showed an early downregulation on day 3 in all inflammatory groups, and a significantly lower expression in the 5- and 10-ng/ml inflammatory groups compared to the control group on day 7. For pro-inflammation-related genes, IL-6 was significantly upregulated in the 5- and 10-ng/ml inflammatory groups on day 7. IL-8 exhibited a remarkable increase in all inflammatory groups except the 1-ng/ml inflammatory group on day 3. COL10 expression declined in inflammatory groups on day 7.

### NO and GAG Release

**FIGURE 5:**
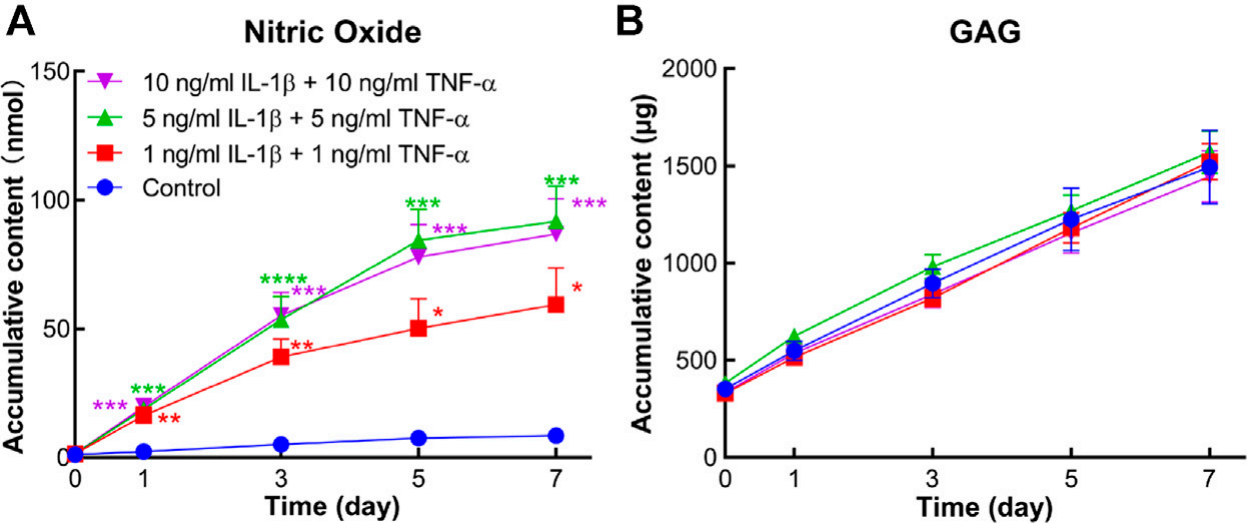
Accumulative amount of nitric oxide (NO) and sulfated glycosaminoglycan (GAG) in the conditioned medium of human osteochondral explants stimulated by different concentrations of IL-1β and TNF-α. **(A)** Accumulative amount of NO in the control group and cytokine-stimulated groups (*n* = 6–11). **(B)** Accumulative amount of GAG in the above groups (*n* = 6–11). Statistical analysis at each time point was performed by one-way analysis of variance (ANOVA) or Kruskal–Wallis test. Data presented as means +SEM. *p < 0.05, **p < 0.01, ***p < 0.001, ****p < 0.0001 vs. control group.

The conditioned medium of each explant was collected at each medium change. Accumulative amounts of NO, a marker reflecting the inflammation microenvironment, were determined in supernatant medium. From day 1 on, accumulative NO levels were significantly higher in all cytokine-induced groups compared to the control group (Figure 5A). In contrast, GAG release in the conditioned medium did not show any difference between the groups with or without stimulation by inflammatory factors (Figure 5B).

### IL-6 and IL-8 Release

**FIGURE 6:**
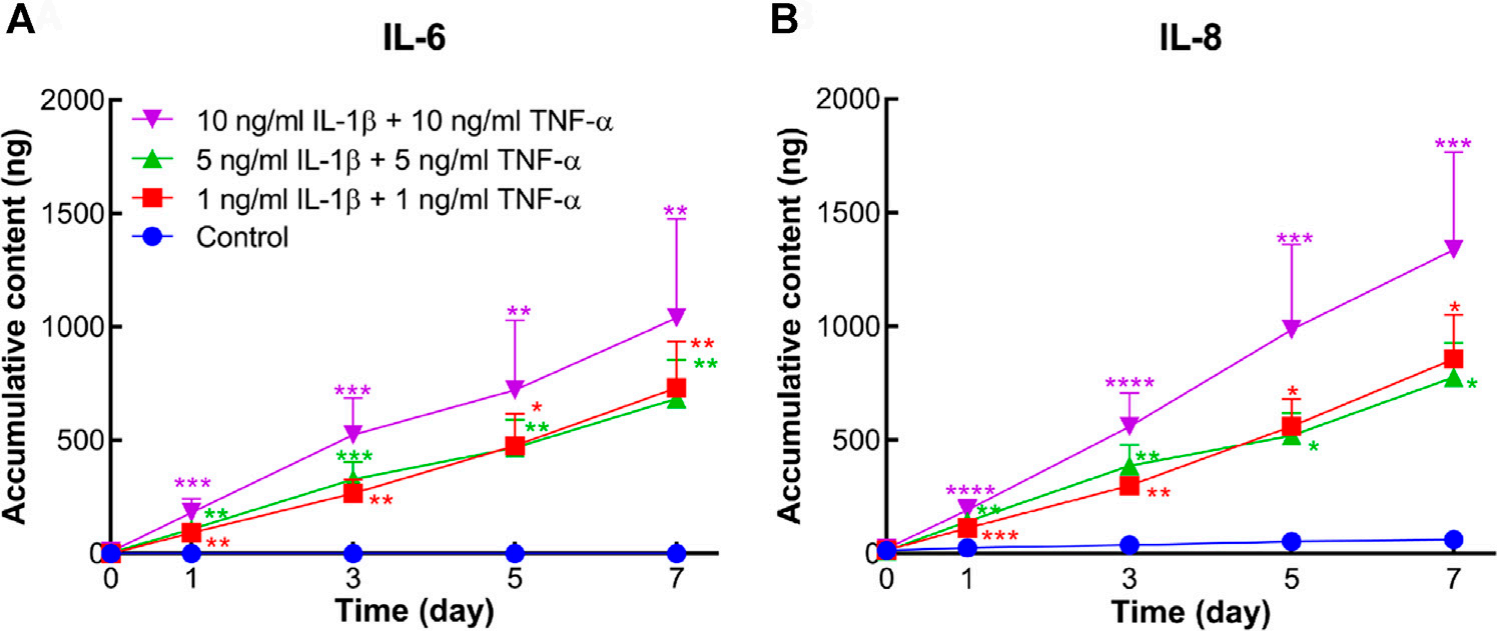
Accumulative amount of IL-6 and IL-8 released into the conditioned medium of human osteochondral explants stimulated by different concentrations of IL-1β and TNF-α. **(A)** Accumulative amount of IL-6 in the control group and cytokine-stimulated groups (*n* = 6–10). **(B)** Accumulative amount of IL-8 in the above groups (n = 6–10). Statistical analysis at each time point was performed by the Kruskal–Wallis test. Data presented as means +SEM. *p < 0.05, **p < 0.01, ***p < 0.001, ****p < 0.0001 vs. control group.

Protein levels of IL-6 and IL-8 secreted into the medium were measured by ELISA. In comparison to the control group, accumulative contents of IL-6 and IL-8 were markedly increased in all inflammatory groups from day 1 to day 7 (Figure 6).

### Summary of the Dose-Dependent Effect of IL-1β and TNF-α on Human Osteochondral Explant Culture

To clearly delineate the effect of the dose and culture period, all the quantitative measurement results are summarized in Table 3, including gene expression data, inflammatory molecule release in conditioned medium, and semiquantitative Safranin O staining evaluation. For the 3-day culture period, most cartilage-related gene expression levels were not altered other than PRG4 and IL-8, although inflammatory indicators in conditioned medium were upregulated and proteoglycan degradation was significantly enhanced. For the 7-day culture duration, the changes in the cartilage were obviously significant from aspects of transcription level as well as medium and histological analysis. Moreover, a dose-dependent correlation was presented between gene expression and concentrations of inflammatory cytokines. With 1 ng/ml IL-1β and 1 ng/ml TNF-α, no changes were observed in gene expressions of ACAN, PRG4, MMP1, and IL6, while the concentrations at 5 and 10 ng/ml induced similarly significant results.

**TABLE 3:** Summary of results in the ex vivo inflammatory OA model.

	Groups	Gene expression								ELISA		NO	Safranin O staining
		ACAN	PRG4	COL2	COL10	MMP1	MMP3	IL-6	IL-8	IL-6	IL-8		
Day3	Control												
	1 ng/ml (IL-1β+TNF-α)		↓							↑	↑	↑	
	5 ng/ml (IL-1β+TNF-α)		↓						↑	↑	↑	↑	
	10 ng/ml (IL-1β+TNF-α)		↓				↑		↑	↑	↑	↑	↓
Day7	Control												
	1 ng/ml (IL-1β+TNF-α)			↓	↓		↑		↑	↑	↑	↑	
	5 ng/ml (IL-1β+TNF-α)	↓	↓	↓	↓	↑		↑	↑	↑	↑	↑	↓
	10 ng/ml (IL-1β+TNF-α)	↓	↓	↓	↓	↑		↑	↑	↑	↑	↑	↓

“↓” means significant downregulation vs. control group; “↑” means significant upregulation vs. control group.

## Discussion

Through encouraging research and development, several potential DMOADs have been found in experimental OA models based on animal studies (Cai et al., 2021). However, their translation to clinical practice is still challenging due to species variances. Cartilage thickness and anatomy of knee/stifle joints vary widely between human and animals (McCoy, 2015). Seok et al*.* found that genomic responses in mouse models poorly simulated inflammatory diseases in humans (Seok et al., 2013). Establishing an appropriate OA model that could accurately mimic human OA phenotypes is indispensable and invaluable for OA drug development. Given that low-grade inflammation is pivotal in OA initiation and development (Robinson et al., 2016), we sought to establish an *ex vivo* inflammatory OA model with specimens from the human femoral head, which were stimulated by different concentrations of IL-1β in combination with TNF-α. Our results showed that tissue viability could be maintained during the culture period. Cartilage of these explants presented a similar inflammatory response like in human OA joints with respect to findings of histology, gene expression, and analysis of supernatant conditioned medium (Martel-Pelletier et al., 2016; Robinson et al., 2016; Boffa et al., 2020).

Most osteochondral explants previously used as *ex vivo* models have originated from animal joints (Maglio et al., 2019), which could only partially represent the response of human cartilage in joint diseases. It would be ideal to have healthy osteochondral tissue as a control group. However, due to the limited access to healthy human osteochondral explants, this was not feasible in the current study. In this work, only the osteochondral tissue with intact cartilage surface was extracted from the femoral head. For the same donor, one or two explants were disposed into each experimental group to minimize the variation among donors. Furthermore, a single stimulation by IL-1β or TNF-α has represented the most popular method to induce an *in vitro* or *ex vivo* OA-like condition (Johnson et al., 2016; He et al., 2020). However, IL-1β and TNF-α seem to be equivalently important during OA progression (Loeser et al., 2016; Robinson et al., 2016). IL-1β and TNF-α in combination are expected to induce a more extensive response of cartilage cells, which should mimic a microenvironment more similar to the OA joints. Since their combination has not been tested before in an inflammatory *ex vivo* model, different concentrations (1, 5, and 10 ng/ml) and culture durations (3 and 7 days) were assessed in our study to compare their effect on human osteochondral explant cultures. In terms of histology results and conditioned medium analysis, all cytokine-stimulated groups showed different levels of OA-like response at both day 3 and day 7. Furthermore, the Safranin O-negatively stained area in histology showed significant differences among different treatment groups on day 7. As for gene expression, the findings presented a large variance among different donors. This may be inevitable for human samples because each donor owned different age, gender, health condition, and Kellgren–Lawrence OA classification in the hip. For the 3-day culture, PRG4 was the sole gene with a remarkable change in all inflammatory groups. PRG4, also known as lubricin, is predominantly produced by superficial zone chondrocytes to protect the cartilage surface from damage (Li et al., 2007; Jay and Waller, 2014). In our model, chondrocytes in the superficial zone of explants were more vulnerable to inflammatory stimulation supplemented in the medium. Thus, the PRG4 gene expression presented an early downregulation. Groups stimulated by 1 ng/ml IL-1β and 1 ng/ml TNF-α still showed remarkable changes in anabolic gene (COL2), catabolic gene (MMP3), and inflammatory gene expression (IL-8) on day 7. These results suggested that the combination of 1 ng/ml IL-1β and 1 ng/ml TNF-α was sufficient to induce a certain grade of inflammatory and catabolic effect on human osteochondral explants after 7 days of culture. Furthermore, inflammatory cytokines at the concentration of 5 ng/ml demonstrated more profound inflammatory and catabolic effects, as indicated by significant downregulation of ACAN and PRG4 gene expression, upregulation of MMP1 and IL6 gene expression, and depletion of proteoglycan in the cartilage layer. Inflammatory cytokines at the concentration of 10 ng/ml showed comparable results as the 5 ng/ml group. These results indicated that IL-1β and TNF-α at 1 ng/ml may be used to induce a mild inflammatory OA condition, whereas the concentrations of 5 and 10 ng/ml mimic a severe inflammatory OA condition on the human osteochondral explant.

The expression of COL10, a chondrocyte hypertrophy marker (van der Kraan and van den Berg, 2012), was previously reported to be upregulated in OA cartilage (He et al., 2019). However, a decrease in COL10 expression in inflammatory OA groups was observed in the current study. Most research data demonstrating upregulation of COL10 in OA cartilage were based on *in vivo* studies rather than *ex vivo* experiments. This discrepancy on COL10 expression in OA condition may be due to the species or model differences, which need to be further elucidated in future studies. MMP13, ADAMTS4, and ADAMTS5 were all reported to be elevated in OA (Malemud, 2019), but remained unchanged in our study (Supplementary Figure S1). We considered the reason to be that the baseline of these genes in the explants may already have been too high to induce remarkable changes within such short stimulation duration. The unchanged expression of these genes, mainly responsible for disintegrating GAG in cartilage extracellular matrix (Yang et al., 2017; Malemud, 2019), may partially explain the similar GAG amount released into conditioned medium of untreated and cytokine-treated groups. Additionally, there may exist differences in the detection methods between Safranin O staining and DMMB. Safranin O staining can reveal local proteoglycan degradation difference in a small region (superficial zone of the cartilage), while small differences of released GAG into the conditioned medium may not be detectable by the DMMB method. If GAG catabolically released in the medium was comparable in all groups, the proteoglycan in the cartilage after cytokine treatment could also be decreased because the anabolic gene expression level (ACAN) was also downregulated in the treated groups. Houtman *et al.* also reported unchanged GAG amounts in all groups of human osteochondral explants after the 6-day treatment, but a significant elevation among different groups from day 10 (Houtman et al., 2021). These results indicated that a longer culture period may be needed to observe a difference in GAG release in our model.

There are some limitations in the current study. One weakness of the proposed model concerns the scalability, donor variability, and dependency of patients undergoing joint replacement surgery. Another limitation of this work is that it focuses on osteochondral tissue responses in an artificial setting. The role of mechanical loading, angiogenesis, or cross talk with synovial tissue and fluid cannot be considered under the described culture conditions. The influence of synovial inflammation on osteochondral tissues could, however, be investigated by coculture experiments or stimulation with conditioned medium. Adaptation of the explant model to a mechanical loading bioreactor commonly used for 3D-tissue engineering constructs (Li et al., 2010) might enable the evaluation of tissue responses under physiological and pathological joint loading conditions.

In our current study, inflammatory cytokines were added at every medium change. For longer culture periods of more than 7 days, we also suggest this method in order to achieve stable osteoarthritic conditions. One of our previous studies (Du et al., 2020) showed that the inflammatory phenotype of human cartilaginous tissue cells would be mitigated in the group of TNF-α stimulation for 24 h followed by basal medium without TNF-α for 24 h, in comparison to the group of TNF-α stimulation for 48 h. For drug screening using our model, intervention with drugs at the beginning can be performed to assess the anti-inflammatory or inflammation prevention effects of drugs at the early phase of OA, while treatment with drugs after 7 days can evaluate their therapeutic effects on midterm or end-stage OA. The time points of drug application can be selected according to the aim of the specific study.

The inflammatory OA *ex vivo* model established in this study will have many applications in OA research. Firstly, OA-related genes or proteins, discovered by animal studies, could be tested with human osteochondral explant samples to evaluate their clinical relevance. Secondly, novel signaling pathways associated with OA disease mechanisms can be studied. Small molecules or drugs which are potential DMOADs could be evaluated using this preclinical explant model. Future research may include the analysis of the subchondral bone to investigate the interplay between cartilage and bone tissue in OA progression. The osteochondral explants can also be cocultured with synovium or fat tissue to explore the mechanism of OA as a joint disease in a more complex setting. Last but not least, physical stimulations like mechanical loading imposed by bioreactors could be substitutes of cytokines to mimic other clinical OA subtypes or investigated as complementary therapeutic measure (Ossendorff et al., 2018).

## Conclusion

An *ex vivo* inflammatory OA model was established with explants from the human femoral head by stimulation with IL-1β in combination with TNF-α at a low concentration. The inflammatory OA model could be utilized as a preclinical tool to uncover the underlying OA mechanism and screen new drugs for treatment as there is no species barrier for clinical translation.

## Data Availability

The original contributions presented in the study are included in the article/Supplementary Material; further inquiries can be directed to the corresponding author.
