# Supplementary material for: Establishment of an Ex Vivo Inflammatory Osteoarthritis Model With Human Osteochondral Explants
Source: Front Bioeng Biotechnol. 2021 Dec 21;9:787020. doi: 10.3389/fbioe.2021.787020 (PMC8724558; doi:10.3389/fbioe.2021.787020)
Supplement: Supplementary file 1 [file DataSheet1.docx]

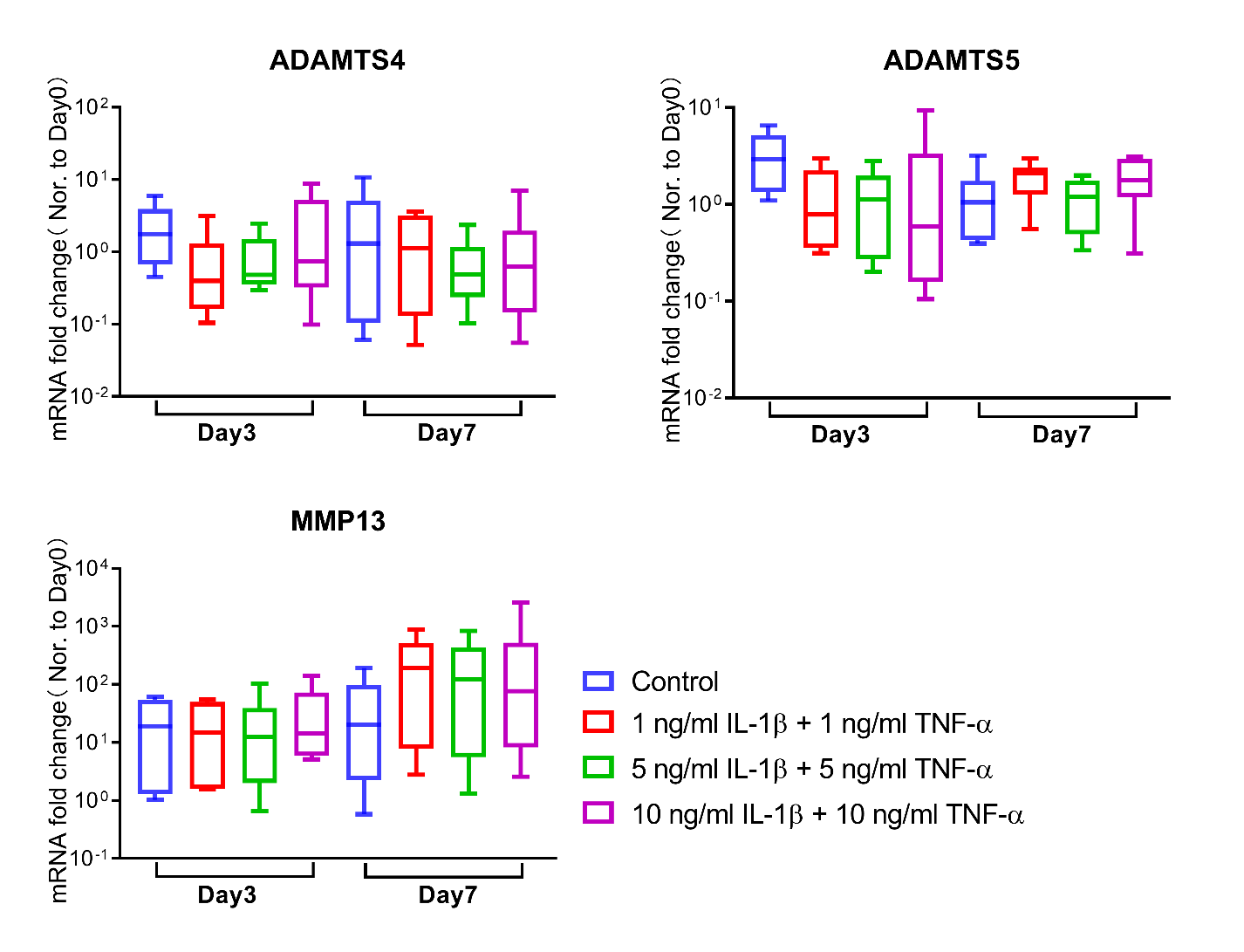
Supplementary Figure 1. Gene expression of MMP13, ADAMTS4, and ADAMTS5 of chondrocytes in osteochondral explants from human femoral heads stimulated with different concentrations of IL-1β and TNF-α for 3 days and 7 days, which was measured by qRT-PCR (n = 5-7). Statistical analysis was performed by one-way analysis of variance (ANOVA). Data presented as box and whiskers plots with boxes indicating the 25^th^ to 75^th^ percentiles, whiskers indicating the maximum and minimum values, horizontal lines in the boxes indicating medians.
